# Bioimaging insights into structural pathways of cell-to-cell communication within the male (MGU) and female (FGU) germ units of *Arabidopsis thaliana*

**DOI:** 10.1007/s00299-025-03441-w

**Published:** 2025-02-14

**Authors:** Wiktoria Parzych, Kamila Godel-Jędrychowska, Michał Świdziński, Janusz Niedojadło, Ewa Kurczyńska, Katarzyna Niedojadło

**Affiliations:** 1 Department of Cellular and Molecular Biology, Faculty of Biological and Veterinary Sciences, Nicolaus Copernicus University, Torun, Poland; 2 Institute of Biology, Biotechnology and Environmental Protection, Faculty of Natural Sciences, University of Silesia, Katowice, Poland

**Keywords:** Pollen, Embryo sac, Male germ unit (MGU), Female germ unit (FGU), Sperm cell, Egg cell, Central cell, Cell-to-cell communication

## Abstract

**Key message:**

Cytoplasmic connections are present between cells within male and female germ units (MGU, FGU), suggesting potential structural pathways for communication.

**Abstract:**

Cell-to-cell communication within the male germ unit (MGU), which consists of two sperm cells and the vegetative cell nucleus, and the female germ unit (FGU), comprising the synergids, the egg cell, and the central cell, is crucial for gamete maturation, fertilization, and early embryogenesis in angiosperms. The MGU facilitates the transport and delivery of immotile sperm cells via the elongating pollen tube to the FGU/embryo sac, which is deeply embedded within the ovule and the ovary. Through applying various bioimaging techniques at both electron and light microscopy levels, we examine the structure and the function of these units in the model plant *Arabidopsis thaliana*, with a particular focus on potential structural pathways for communication. In the MGU, this communication is facilitated by a cytoplasmic projection that connects the sperm cells to the lobed vegetative nucleus. In the FGU, the extracellular matrix adjacent to the egg cell, central cell, and synergids plays a similar role. We discuss our findings in the context of previous studies on *Hyacinthus orientalis*, where, in contrast to *Arabidopsis*—which possesses a tricellular pollen structure—sperm cells are formed within the growing pollen tube.

**Supplementary Information:**

The online version contains supplementary material available at 10.1007/s00299-025-03441-w.

## Introduction

The life cycle of angiosperms includes two distinct phases, the vegetative phase (sporophyte) and the generative phase (gametophyte). During the transition from the vegetative to the generative phase (from 2 to 1n), somatic cells within the male and female reproductive organs—anthers and pistils with ovules—differentiate into spore mother cells. These cells undergo reduction division and initiate gametophyte development. In the anther, microspore mother cells (MiMC) are surrounded by somatic tapetum cells, whereas only one megaspore mother cell (MMC) differentiates per ovule. As a result of the MiMC meiosis, a tetrad of haploid microspores is formed (microsporogenesis). In turn, the MMC divides to form four linearly arranged megaspores (megasporogenesis), and only one haploid megaspore survives to become the functional megaspore, while three others degenerate. The subsequent stage involves the development of microspores into male gametophytes—pollen grains (microgametogenesis) and the development of the functional megaspore into the female gametophyte—the embryo sac (megagametogenesis) (Drews et al. 1998; Yadegari and Drews 2004; Borg et al. 2009; Twell 2011).

The microspore is the first cell of the haploid generation from which the male gametophyte, i.e., the pollen grain, will develop. The asymmetric division of the microspore (mitosis I) leads to the formation of two functionally differentiated cells—a generative cell (GC) directed toward the reproductive pathway and a vegetative cell (VC) which performs somatic functions. In most flowering plants, pollination occurs in the two-celled pollen grain stage. The GC (mitosis II) division into two sperm cells (SCs) occurs within the growing pollen tube. However, in some dicotyledonous plants, such as those in the Cruciferae family and the monocotyledonous grasses, the GC divides into two SCs (three-celled pollen) during pollen grain maturation (Honys et al. 2006; Twell 2011; Gómez et al. 2015; Hafidh et al. 2016). After pollination, the VC grows into a pollen tube, transporting the male gametes to the embryo sac (Heslop-Harrison 1987; Mascarenhas 1993; Taylor and Hepler 1997). In most flowering plants (e.g., *Arabidopsis*, *Zea*, *Oryza*), the embryo sac develops from a single reduced megaspore, a process known as monosporic development. Three mitotic divisions of the functional megaspore produce an eight-nucleate embryo sac, which subsequently organizes into a seven-celled female gametophyte of the *Polygonum* type (Huang and Russell 1992; Sánchez-León and Vielle-Calzada 2010; Drews and Koltunow 2011). After migration to the micropylar pole and undergoing cellularization, three nuclei form an egg apparatus consisting of an egg cell (EC) and two synergids (S). The Ss produce attractants that guide the pollen tube, and one becomes the site of pollen tube entry and the release of SCs (Higashiyama et al. 2001; Punwani and Drews 2008; Li et al. 2009; Higashiyama and Yang 2017). At the chalazal pole, three nuclei form antipodal cells (A) that perform nutritional functions (Song et al. 2014). The remaining two polar nuclei fuse to form a homodiploid nucleus (secondary nucleus, 2n) within the central cell (CC) (Huang and Russell 1992; Chen et al. 2007; Liu et al. 2010). The process of double fertilization initiates seed formation. The fusion of one SC with the EC, a diploid embryo develops, which will give rise to a new generation of sporophyte (transition from 1 to 2n), whereas the fusion of the second SC with the CC generates the nutritional tissue—triploid endosperm (Weterings and Russell 2004; Hamamura et al. 2011; Dresselhaus et al. 2016).

**Fig. 1:**
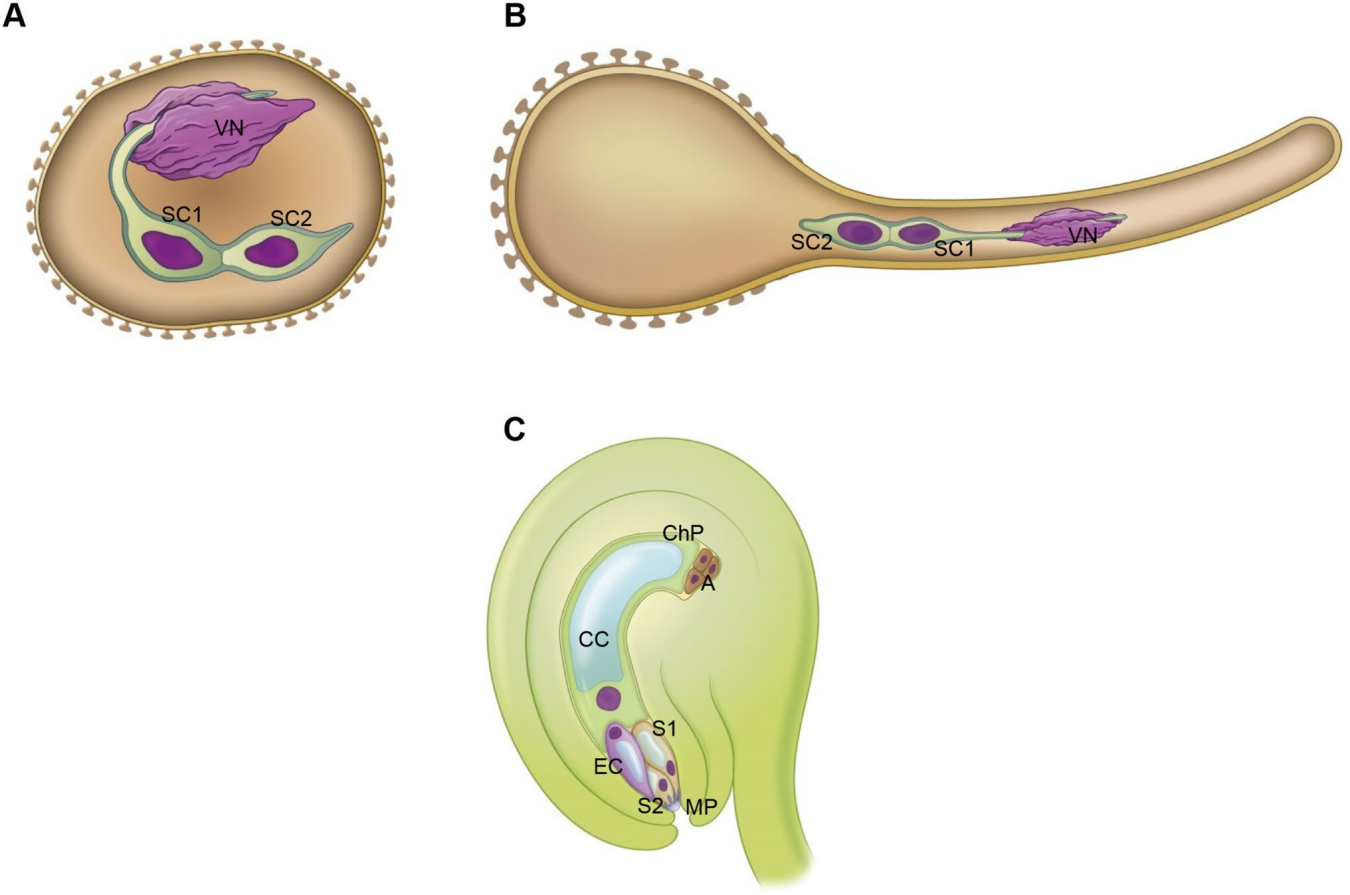
Model of the organization of *A. thaliana* “male germ unit” (MGU) in pollen grain (**A**) and tip-growing pollen tube (**B**) and “female germ unit” (FGU) in the ovule (**C**). *VN* vegetative nucleus, *SC1*, *SC2* sperm cells, *EC* egg cell, *S1*, *S2* synergids, *CC* central cell, *A* antipodal cells, *MP* micropylar pole, *ChP* chalazal pole

In the context of fertilization, the terms “male germ unit” (MGU) and “female germ unit” (FGU) have been proposed (Russell and Cass 1981; Dumas et al. 1984, 1985). The MGU consists of two SCs and the vegetative cell nucleus (VN), while FGU comprises the EC flanked by two Ss (collectively forming “egg apparatus”) and the CC (Fig. 1). The concept of MGU and FGU suggests that these are not only structural units that represent the minimal number of cells required for successful double fertilization but also functional (Russell 1993; Dumas and Mogensen 1993). One SC is consistently associated with the vegetative cell (VC) nucleus via a cytoplasmic projection hooked up to the lobed VN (Jensen and Fisher 1970; Russell 1991; Sprunck 2020) (Fig. 1A). This physical association facilitates the transport of male gametes as passive cargo during the movement of the MGU within the germinal pollen and the growing pollen tube through the floral tissue to reach the embryo sac (Zhou and Meier 2014; Vogler et al. 2015; Zhang et al. 2017) (Fig. 1B). Additionally, this connection ensures cell-to-cell communication and information exchange between cells (Slotkin et al. 2009; McCue et al. 2011). The components of the FGU (Fig. 1C) attract and receive the pollen tube, release the sperm cells, and transport them toward fusion with female gametes (Russell 1993; Dumas and Mogensen 1993). Intercellular communication within FGU also plays a crucial role in these processes.

Understanding the unique structure and function of the MGU and FGU is essential for elucidating the mechanisms underlying sexual reproduction in angiosperms. In this study, we analyzed the male and the female gametophyte cells of *Arabidopsis thaliana*. Using light and electron microscopy bioimaging techniques, we focused on the close contact between cells within the units, particularly on the extracellular matrix with the potential intercellular connections that facilitate communications between cells: a potential symplasmic connection between the EC and the CC and the physical association of one SC to the VN in pollen grain and in vitro growing pollen tube.

## Material and methods

*Arabidopsis thaliana* ecotype Col-0 and *Hyacinthus orientalis* (a commercial cultivar) plants were cultivated in a greenhouse under controlled conditions, with a 16 h light/8 h dark cycle at 21 °C and 60% humidity.

### Ultrastructure analysis

For transmission electron microscopy (TEM) analysis*,* en bloc staining with lead aspartate method was used (Walton 1979; Kopriwa 1984). On the day of anthesis, 40 flowers were transferred into a 1.5 ml tube containing 1 ml of a fixation solution containing 3% (v/v) glutaraldehyde (Polysciences Europe GmbH, Germany) in 0.15 M sodium cacodylate buffer pH 7.4 or immediately submerged in 1 ml of germination medium containing 5 mM CaCl_2_·2H_2_O, 0,01% (w/v) H_3_BO_4_, 5 mM KCl, 1 mM MgSO_4_·7H_2_O, 10% (w/v) sucrose (Merck KGaA, Darmstadt, Germany) pH 7.5 for 30 min according to the method described by Boavida and McCormick (2007) and Dardelle et al. (2010) to hydrate the pollen. The tubes were then vortexed to release the pollen grains. After removing the flowers, the pollen suspension was pelleted down at 3200 g for 6 min. A fresh 250 µl of fixation solution was added to the pellet. For pollen tubes cultivation, 250 µl of the germination medium was added to the hydrated pollen pellet. The pollen grains were transferred to glass vials (14 × 45 mm) and incubated in a humid chamber in the dark at 22 °C. After 6 h of cultivation, the pollen tubes were washed with 0.15 M sodium cacodylate buffer pH 7.4, and then the fixation solution was added. All samples were fixed for 12 h at 4 °C. Then, the pollen tubes were adhered to small pieces of Thermanox^®^ plastic sheet (Ted Pella) coated with 0.1% Poly-L-lysine (Merck KGaA, Darmstadt, Germany). For ultrastructure analysis of the FGU, pistils from emasculated flowers were dissected under a stereomicroscope on the day of anthesis and immediately transferred to the fixation solution for 12 h at 4 °C.

All fixed materials (pollen, pollen tubes, and pistils) were washed in 0.15 M sodium cacodylate buffer pH 7.4 and double post-fixed in 2% (v/v) OsO_4_ (Polysciences Europe GmbH, Germany) with 3.0% potassium ferricyanide (w/v) in 0.15 M sodium cacodylate buffer pH 7.4 (1 h on ice) followed in by 1% (w/v) thiocarbohydrazide (Merck KGaA, Darmstadt, Germany) (at RT for 20 min) and then 2% (v/v) OsO_4_ (at RT for 30 min). The samples were stained overnight with 1% (w/v) uranyl acetate and then treated for 30 min at 60 °C in lead aspartate solution pH 5.5. After each incubation step, the material was rinsed three times with Milli-Q-filtered water. Serial dehydration of the material was performed in graded ethanol series and then embedded in Spurr resin (Polysciences Europe GmbH, Germany) according to the standard protocol. Ultrathin sections were collected on copper grids and examined by transmission electron microscopy (Jeol EM 1010) at 80 kV.

### FISH reactions

For in situ hybridization, *A. thaliana* pollen and pollen tubes in the germination solution were mixed with a fixation medium containing 100 mM PIPES buffer pH 6.9, 4 mM MgSO_4_·7H_2_O, 4 mM EGTA, 10% (w/v) sucrose and 5% (v/v) formaldehyde (Polysciences Europe GmbH, Germany) and incubated for 90 min at room temperature. Following fixation, the samples were rinsed three times by centrifugation with 50 mM PIPES buffer pH 6.9, 2 mM MgSO_4_·7H_2_O, and 2 mM EGTA (Merck KGaA, Darmstadt, Germany) and three times in PBS pH 7.2 (Dardelle et al. 2010) and saline-sodium citrate buffer pH 7.0 (4xSSC). The FISH reaction was also performed on *H. orientalis* pollen tubes after the GC division into two SCs. Freshly collected mature pollen grains were used for germination in the Brewbaker and Kwak (1963) medium with 10% (w/v) polyethylene glycol 4000 (Merck KGaA, Darmstadt, Germany) modified by adding pistils from the pollinated flowers (according to the method described by Zienkiewicz et al. 2011). Cultivation was carried out at 25 °C in the dark and pollen tubes were collected after 24 h of growth and fixed in a mixture of 4% (v/v) paraformaldehyde and 0.5% (v/v) glutaraldehyde (Polysciences Europe GmbH, Germany) prepared in PBS pH 7.2, overnight at 4 °C. The material was then enzymatically digested in a mixture of 1% cellulase R10 (Serva Electrophoresis GmbH, Heidelberg, Germany) and 27 U pectinase (Merck KGaA, Darmstadt, Germany) in 0.01 M citrate buffer pH 4.8 for 25 min at 37 °C and next rinsed in 0.01 M citrate buffer pH 4.8 and saline-sodium citrate buffer pH 7.0 (4xSSC).

*A. thaliana* pistils from emasculated flowers were dissected on the day of anthesis and fixed in a solution containing 4% formaldehyde (v/v) and 0.25% (v/v) glutaraldehyde (Polysciences Europe GmbH, Germany) in PBS pH 7.2 for 24 h at 4 °C. The fixed material was dehydrated through a graded series of ethanol containing 10 mM dithiothreitol (DTT, Merck KGaA, Darmstadt, Germany) and subsequently supersaturated. It was embedded in BMM resin (butyl methacrylate, methyl methacrylate, 0.5% benzoyl ethyl ether with 10 mM DTT, Merck KGaA, Darmstadt, Germany) at – 20 °C under UV light for polymerization. Semithin sections were mounted on Polysine®Slides (Thermo Fisher Scientific Inc, Waltham, MA, USA). Before the FISH reaction, the resin was removed with two changes of acetone, and then the material was rinsed twice in water and finally in PBS pH 7.2 and 4xSSC pH 7.0 (Niedojadło et al. 2012, 2015a, b).

Poly(A) RNA was detected using a DNA oligonucleotide probe (5′Cy3 T(T)29, Genomed, Warsaw, Poland) resuspended in hybridization buffer (30% [v/v] formamide, 4 × SSC, 5 × Denhardt’s buffer, 1 mM EDTA, 50 mM phosphate buffer) to a concentration of 50 pmol/ml. Hybridization was performed 12 h at 26 °C. After labeling, the material was stained with Hoestch 33,342 in PBS pH 7.2 (1:1000) for DNA detection and mounted in ProLong Gold Antifade reagent (Thermo Fisher Scientific Inc, Waltham, MA, USA).

Images of pollen and pollen tubes were registered with a Leica TCS SP8 confocal laser scanning microscope using a diode 405 laser and a diode laser DPSS 561. Optimized pinhole, long exposure (200 Hz), and 63x (numerical aperture 1.4) Plan Apochromat DIC H oil immersion lens were used. The images were collected sequentially in the blue (Hoechst 33342, PMT detector with sensitivity range of 420–470 nm) and red (Cy3, PMT detector with sensitivity range of 570–610 nm) channels using low laser power (1.5% of maximum power) and sequential collection to minimize bleed-through between the channels. The obtained data were corrected for background autofluorescence as determined by negative control signal intensities. Images of semithin sections of *A. thaliana* pistils were acquired using an Olympus BX50 fluorescence microscope. The UPlanFI 100 × (N.A. 1.3) oil immersion lens and narrow band filters (U-MNU, U-MNG) were used. The results were registered with an Olympus XC50 digital color camera and Cell^B^ software (Olympus Soft Imaging Solutions GmbH, Münster, Germany).

### Immunocytochemistry

The pistils of *A. thaliana* were prepared for embedding in BMM resin (butyl methacrylate, methyl methacrylate, 0.5% benzoyl ethyl ether with 10 mM DTT (Merck KGaA, Darmstadt, Germany) according to Niedojadło et al. (2015a, b). The material was cut into semithin sections. (1.5 µm) and collected on Thermo Scientific™ Polysine adhesion microscopic slides (Thermo Fisher Scientific Inc, Waltham, MA, USA). Before the reaction, the resin was removed using acetone, and the material was washed in distilled water and PBS pH 7.4. After incubation with 2% BSA (bovine serum albumin) in PBS pH 7.4 for 30 min at room temperature, the sections were incubated with anti-pectins rat primary antibodies: JIM7, LM19, and LM5 (Kerafast) diluted 1:20 in 0.2% BSA in PBS pH 7.4 overnight at 4 °C and with AlexaFluor®488-conjugated goat anti-rat secondary antibody (Thermo Fisher Scientific Inc, Waltham, MA, USA) diluted 1:250 in 0.2% BSA in PBS pH 7.4 for 1 h at 37 °C. Additionally, DNA was stained with 4 pg/ml DAPI (4′,6-diamidino-2-phenylindole, Merck KGaA, Darmstadt, Germany) solution in PBS pH 7.4 for 5 min. Next, slides were washed in PBS pH 7.4, dried at room temperature, and mounted with ProLong^TM^Gold antifade reagent (Thermo Fisher Scientific Inc, Waltham, MA, USA). To perform control reactions, the incubations with primary antibodies were omitted. The controls showed no labeling (not shown). Semithin sections were analyzed with an Olympus BX50 fluorescence microscope. UPlanFI 100 × (N.A. 1.3) oil immersion lens and narrow band filters (U-MNU, U-MNB) were used. The results were registered with an Olympus XC50 digital color camera and Cell^B^ software (Olympus Soft Imaging Solutions GmbH, Münster, Germany).

### Methylene blue and Calcofluor White stainings

To investigate the structure of the mature embryo sac, the *A. thaliana* pistils were embedded in BMM resin and cut into semithin sections as described previously. Next, the sections were stained with 1% w/v methylene blue and washed in distilled water. After drying at room temperature, the slides were mounted with DPX Mount for histology (Merck KGaA, Darmstadt, Germany). The fluorochrome Calcofluor White Stain (Merck KGaA, Darmstadt, Germany) composed of Calcofluor White M2R 1 g/L and Evans Blue 0.5 g/L was used for the cytochemical staining of cellulose. The sections were stained for 2 min, washed with distilled water, and then mounted with ProLong^TM^Gold antifade reagent (Thermo Fisher Scientific Inc, Waltham, MA, USA). The stained sections were analyzed using an Olympus BX50 fluorescence microscope with a UPlanFI 100 × (N.A. 1.3) oil immersion lens and narrow band filter U-MNU. The results were registered with an Olympus XC50 digital color camera and Cell^B^ software (Olympus Soft Imaging Solutions GmbH, Münster, Germany).

## Results

### Male germ unit

**Fig. 2:**
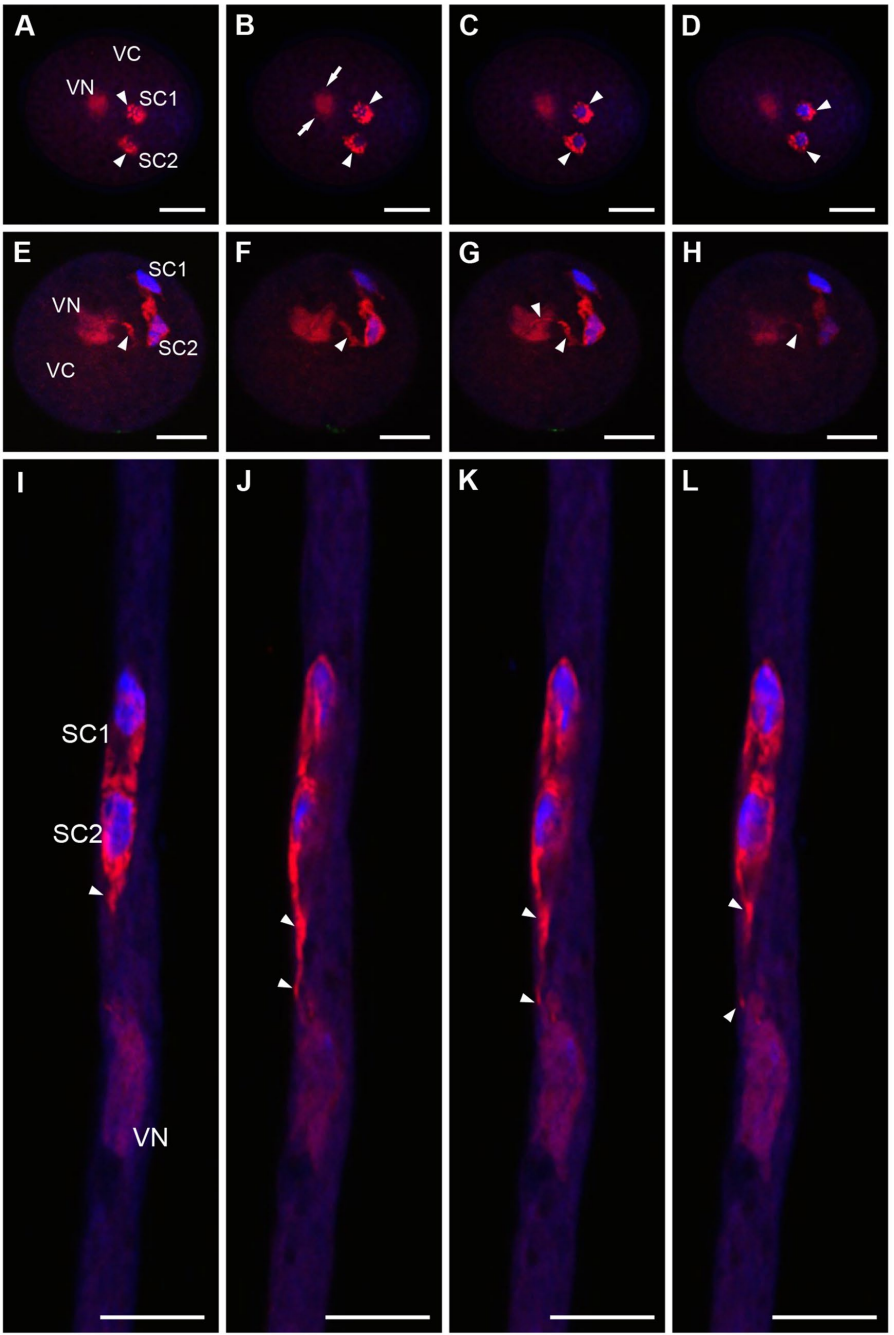
Localization of poly(A)^+^RNA in *A. thaliana* MGU, optical serial sections of the dehydrated pollen grain (**A**–**D**), after pollen rehydration (**E**–**H**) and in vitro growing pollen tube (**I**–**L**). During dehydration in SCs, polyadenylated transcripts accumulate in granules (**A**–**D**, arrowheads), and after rehydration, they are dispersed in the cytoplasm; the pollen cytoplasmic projection is visible (**E**–**H**, arrowheads). After pollen germination, a high level of poly(A) ^+^ RNA is detected in the SCs cytoplasm, and the cytoplasmic connection of male gametes to VN is still observed (I–L, arrowhead*s*). The homogenous signal of the fluorescence is present in VN during all analyzed stages of male gametophyte development. *VC* vegetative cell, *VN* vegetative nucleus, *SC1, SC2* sperm cells; *red* signal of FISH reaction, *blue* Hoechst 33342 staining; bars 5 µm

Fluorescence in situ hybridization (FISH) using an oligonucleotide probe complementary to the poly(A) tail of RNA indicated different spatio-temporal distribution of transcripts in *A. thaliana* male gametophyte cells (Fig. 2). Immediately after anthesis, in dehydrated pollen grains, poly(A)^+^RNA was detected in the cytoplasm of the VC and in both gametes (Fig. 2A–D, ESM_1). The fluorescence signal was homogenously distributed in the nucleus of the VC (VN) and in small clusters around the nucleus in the cytoplasm (Fig. 2B, arrows), while the fluorescence signal in the remaining cytoplasmic area was lower and more dispersed. In SCs, FISH analysis revealed an accumulation of polyadenylated RNA in the cytoplasm with a less homogenous distribution in the nucleus. Serial confocal optical sections showed distinct clusters of fluorescence in the cytoplasm of SCs (Fig. 2A–D, arrowheads). During rehydration, male gametes' poly(A)^+^RNA distribution pattern changed (Fig. 2E–H, ESM_2). Polyadenylated transcripts become dispersed throughout the cytoplasm. Interestingly, the FISH reaction highlighted a cytoplasmic connection between SCs and the VN (Fig. 2E–H, arrowheads). Homogenous poly(A)^+^RNA localization in the MGU remained in in vitro growing pollen tubes (Fig. 2I–L, Supplementary Fig. S1A–C). The level of polyadenylated transcripts was higher in the cytoplasm compared to the nucleus of SCs. The cytoplasmic projection connecting one SC with the VN was strongly detectable (Fig. 2K, Fig. S1A–B, arrowheads).

To verify the universality of poly(A)^+^RNA localization in the SCs formed after the GC mitosis in the growing pollen tube (bicellular pollen grain), we examined *Hyacinthus orientalis* L. as a model. Surprisingly, the distribution pattern of polyadenylated transcripts in male gametophyte cells was consistent with that observed in the SCs of the dehydrated tricellular pollen grain of *A. thaliana* (Fig. S1). In hyacinth, after SCs formation, the MGU exhibits a physical association between SCs and the VN. Within this organized structure, the poly(A)^+^RNA was localized as numerous granules in the cytoplasm of both gametes (Fig. S1D–J, arrowheads). In contrast, the fluorescence signal in the VN remained uniformly distributed (Fig. S1D–J).

**Fig. 3:**
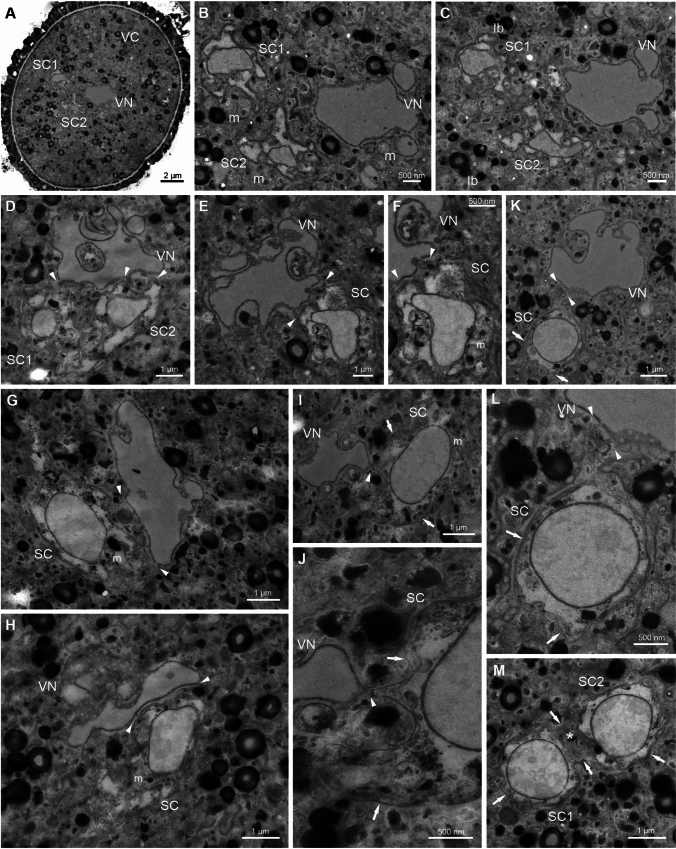
Transmission electron micrographs of sections through MGU of dehydrated *A. thaliana* pollen grain. In the center of the VC MGU formed by VN and two SCs are visible (**A**). SCs are located near the VN (**B**–**D**). SCs are irregular in shape and form protrusions, which provide them with close contact with VN (**E**–**L**, arrowheads); they also are separated from each other by their plasma membranes and enclosed within the peri-germ cell membrane (**J**–**M**, arrows). The SCs connecting area has an interlocking cell border filled with electron-dense material (Fig. 3 M, asterisk). The VN is highly lobbed with numerous nuclear pores (**A**–**I**). *VC* vegetative cell, *VN* vegetative nucleus, *SC1, SC2* sperm cells, *m* mitochondrion, *lb* lipid body

**Fig. 4:**
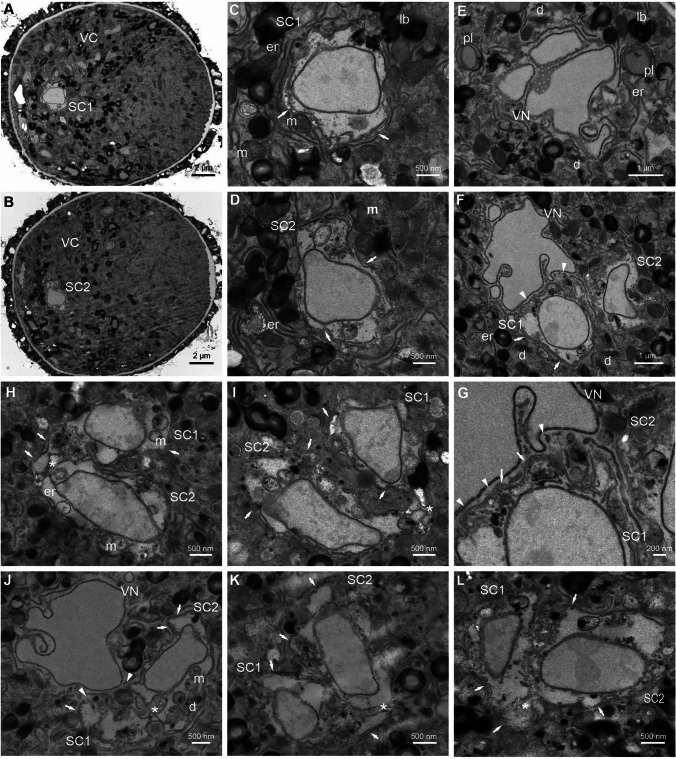
Transmission electron micrographs of sections through MGU of rehydrated *A. thaliana* pollen grain. The membrane structures/organelles are more dispersed and become more visible in VC (**A**, **B**) and SCs (**C**, **D**). VN is still highly lobbed with dispersed chromatin and numerous nuclear pores (**E**). After hydration, the close proximity of VN and SCs becomes more visible (**F**–**G**, arrowheads). The plasma membranes of SCs and peri-germ cell membrane are distinctly discernible (**C**–**D**, **F**–**L**, arrows), and interlocking cell borders connect SCs, which also share a common area filled with electron-dense material (**H**–**L**, asterisks). *VC* vegetative cell, *VN* vegetative nucleus, *SC1, SC2* sperm cells, *er* endoplasmic reticulum, *d* dictyosomes, *pl* plastid, *m* mitochondrion, *lb* lipid body

We subsequently analyzed the ultrastructure of the MGU organization (Figs. 3, 4) using the en bloc method, which produces general staining and significantly enhances the TEM images of membrane structures in cells (Walton 1979; Kopriwa 1984). During the final stages of maturation within the anther and following its dehiscence, tricellular *Arabidopsis* pollen loses water. In the dehydrated pollen grain, organelles are densely packed in the cytoplasm of the VC. The cytoplasmic membranes were not clearly visible (Fig. 3, Fig. S2). Among the ambiguously distinguished membrane structures, mitochondria and numerous vesicles were present (for example, Fig. 3B). Larger vacuoles were not observed, likely due to their disappearance in the dehydrated state. Lipid bodies, visible as dense osmiophilic structures, were dispersed throughout the cytoplasm (e.g., Fig. 3C). The SCs are located adjacent to the VN, forming the MGU in the center of the VC (Fig. 3A). The VN is large, highly lobbed and contains numerous nuclear pores (Fig. 3A–I, Fig. S2). The male gametes are separated from each other by their plasma membranes and are enclosed within the plasma endomembrane of the VC called peri-germ cell membrane (PGCM) (Sugi et al. 2024) (Fig. 3I–M, arrows, Fig. S2). The area connecting the SCs exhibits an interlocking cell border filled with electron-dense material (Fig. 3M, asterisk). The male gametes are irregularly shaped and develop protrusions that facilitate close contact with the VN (Fig. 3D–L, arrowheads). The SCs have a relatively simple ultrastructure, similar to the VC with a shrunken cytoplasm and few membrane structures, such as mitochondria and vesicles (Fig. 3F–I). Single lipid bodies are also observed (e.g., Fig. 3G). The nuclei of SCs are elongated and less lobed than the VN, and their nuclear envelopes contain pores (Fig. 3B–M, Fig. S2).

The hydration of compatible pollen on the stigmatic papilla and the subsequent activation of germination are affected by the passage of water through the germination apertures of the pollen grain, where the intine is more accessible (Heslop-Harrison 1987). In our study, after rehydrating *A. thaliana* pollen grains by submerging them for 30 min in the germination medium (Boavida and McCormick 2007; Dardelle et al. 2010), we observed significant ultrastructural changes in the VC cytoplasm (Fig. 4). Near the site of pollen tube formation, we noted considerable dilution of the cytoplasm. Generally, the membrane structures/organelles in the VC cytoplasm became more dispersed and visible (Fig. 4A, B). Numerous mitochondria, plastids, dictyosomes, and vesicles were present. The fixation of the material in en bloc staining did not allow us to detect ribosomes, but our observations in previous works (Niedojadło et al. 2015a) suggest that the shape of the cisterns indicates a rough reticulum (e.g., Fig. 4C–F). These changes within the MGU were accompanied by the rapid reformation of the VC membrane system following hydration. The VN remained lobed with dispersed chromatin, while the SCs adopted a more regular and elongated shape (Fig. 4E, F, J, Fig. S3). The relatively simple ultrastructure of male gametes becomes apparent, with only a few organelles, such as mitochondria, dictyosomes, and cisternae of the endoplasmic reticulum, being visible (e.g., Fig. 4 H, J). The nuclei of SCs exhibited more condensed chromatin compared to the VN (e.g., Fig. 4H–I, Fig. S3). Following hydration, the proximity of the VN and SCs becomes more visible; serial pollen sections revealed a cytoplasmic extension in the form of a tail from one of the SCs (Fig. 4F, J, arrowheads, Fig. S3). The plasma membranes of both SCs and the VC were distinctly visible (Fig. 4C, D, F–K, arrows), and SCs were observed to share a common area filled with electron-dense material (Fig. 4H–L, asterisks, Fig. S3).

**Fig. 5:**
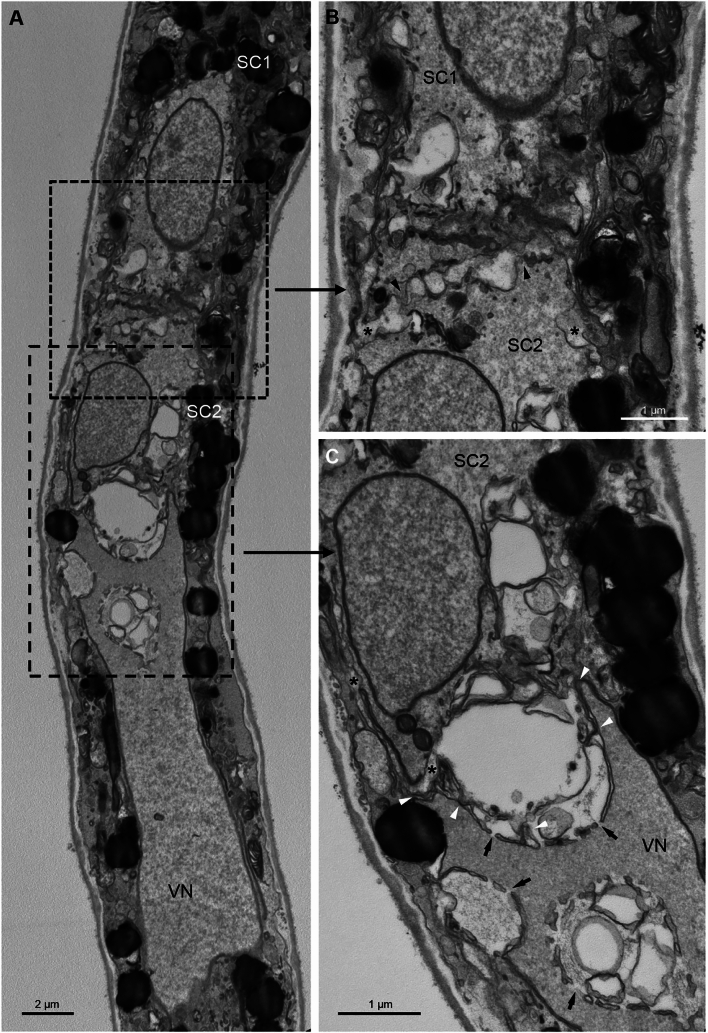
Transmission electron micrographs of sections through MGU of in vitro growing *A. thaliana* pollen tube. The euchromatic VN (**A**) with numerous nuclear pores (**C**, arrows) is lobbed and changes shape to a highly elongated (**A**). Interlocking cell borders connect two SCs (**B**, asterisks), and the plasma membranes of both gametes are in contact with each other (**B**, arrowheads). The tip of one of the SCs, which is in close contact with the VN (C, arrowheads). *VN* vegetative nucleus, *SC1, SC2* sperm cells

In the pollen tube, the SC–VN physical association within the MGU remains evident (Fig. 5). The euchromatic VN is lobed and changed to a highly elongated shape (Fig. 5A) with numerous nuclear pores clearly visible (Fig. 5C, arrows). The spindle-shaped SCs are separated from each other by their respective plasma membranes but remain in close proximity to irregular areas of the extracellular matrix with numerous invaginations, which are the sites of their connection (Fig. 5B, arrowheads). The tip of one SC is in close contact with the VN (Fig. 5C, arrowheads). Within the reduced cytoplasm of the SCs, only a few organelles and vesicles are present. The nuclei of SCs are elongated and contain more condensed chromatin than the VN. Numerous nuclear pores are observed in the nuclear envelope of each SC.

### Female germ unit

**Fig. 6:**
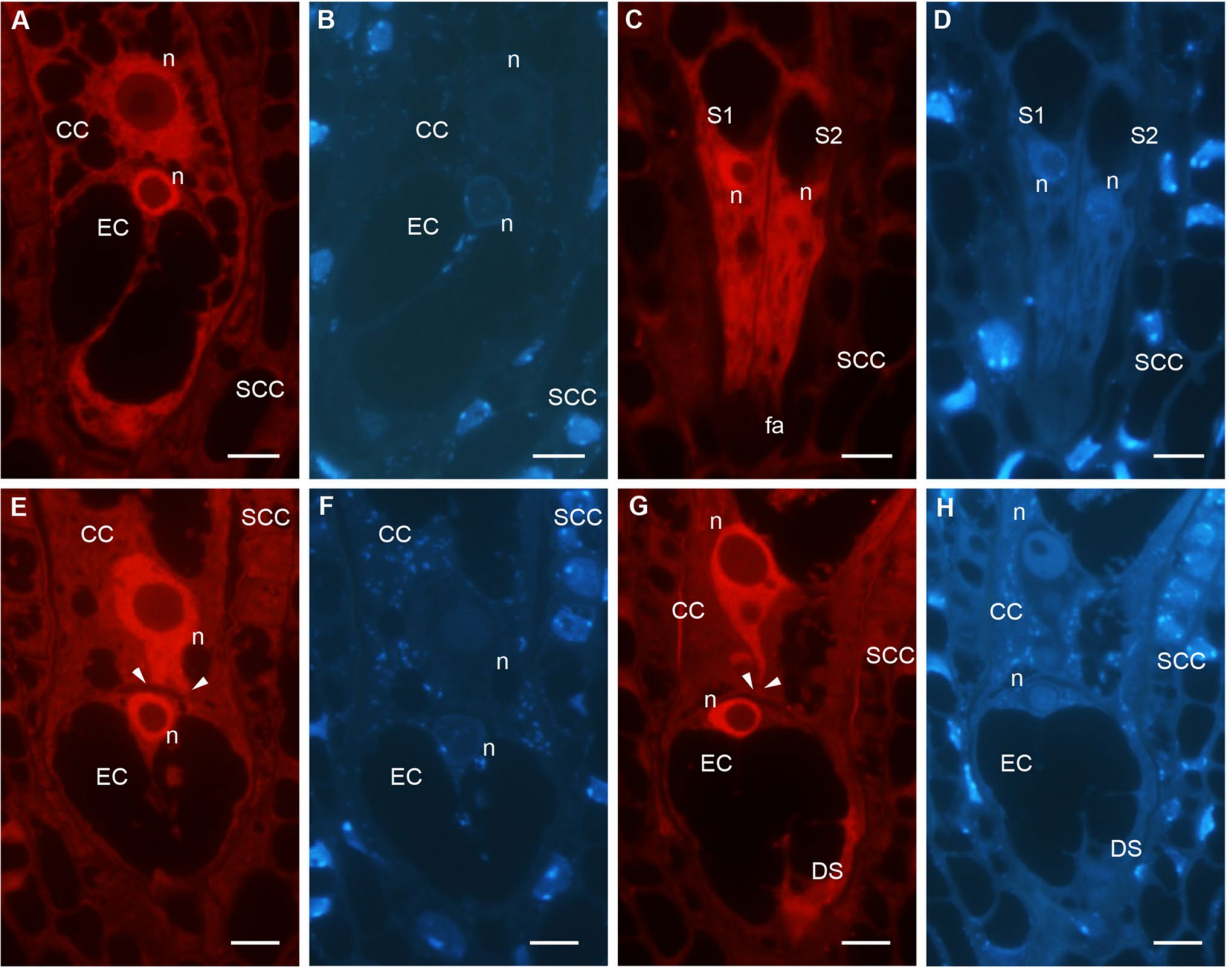
Localization of poly(A)^+^RNA in *A. thaliana* FGU at the different stages of the embryo sac development: FG7 (**A**–**D**), F1 (**E**, **F**) and F2 (**G**, **H**). During anthesis, the highest level of polyadenylated transcript in the nuclei of the female gametes is observed, while in the cytoplasm, the fluorescence signal is lower (**A**, **B**). In the Ss, the poly(A) ^+^RNA is high in both the nucleus and the cytoplasm; the signal is not visible in the area of the filiform apparatus (**C**, **D**). The level and distribution of poly(A)^+^RNA do not change in the following stages when the CC nucleus elongates (**E**, **F**) and after its fertilization (**G**, **H**). During this period, the high poly(A) ^+^RNA level in all the EC and CC nuclei areas, which are close to each other (arrowheads), is still detected. *EC* egg cell, *CC* central cell, *S1, S2* synergids, *DS* degenerative synergid, *SCC* somatic cells, *n* nucleus, *fa* filiform apparatus, A, C, E, G signal of FISH reaction, B, D, F, H DAPI staining; bars 5 µm

Analysis of poly(A)^+^ RNA distribution in mature embryo sacs revealed the highest levels of polyadenylated transcripts in the nuclei of the EC, CC, and Ss compared to the somatic cells of the ovule (Fig. 6A, C, E, G). At stage FG7 (Christensen et al. 1997), when the nucleus of the EC is highly polarized toward its chalazal end, and the nucleus of the CC is in close proximity to the EC (Fig. 6A), the highest level of poly(A)^+^RNA among all cells within the FGU is localized in the cytoplasm of the Ss (Fig. 6C). As development progresses to the FG8/F1 (Christensen et al. 1997), the EC remains polarized while the CC nucleus elongates along the micropyle–chalaza axis. During this stage, poly(A)^+^RNA levels remain high in the nuclei of the female gametes, which are closely positioned to each other (Fig. 6E, arrowheads). At the early stage F2 (Christensen et al. 1997), following pollination, when the three nucleoli are visible in the CC, high levels of poly(A)^+^RNA are still observed in the nuclei of the EC and CC. In the serial optical sections of the nucleus of the primary endosperm cell, the contact by the nuclear protrusion directed toward the EC is visible (Fig. 6G, arrowheads).

**Fig. 7:**
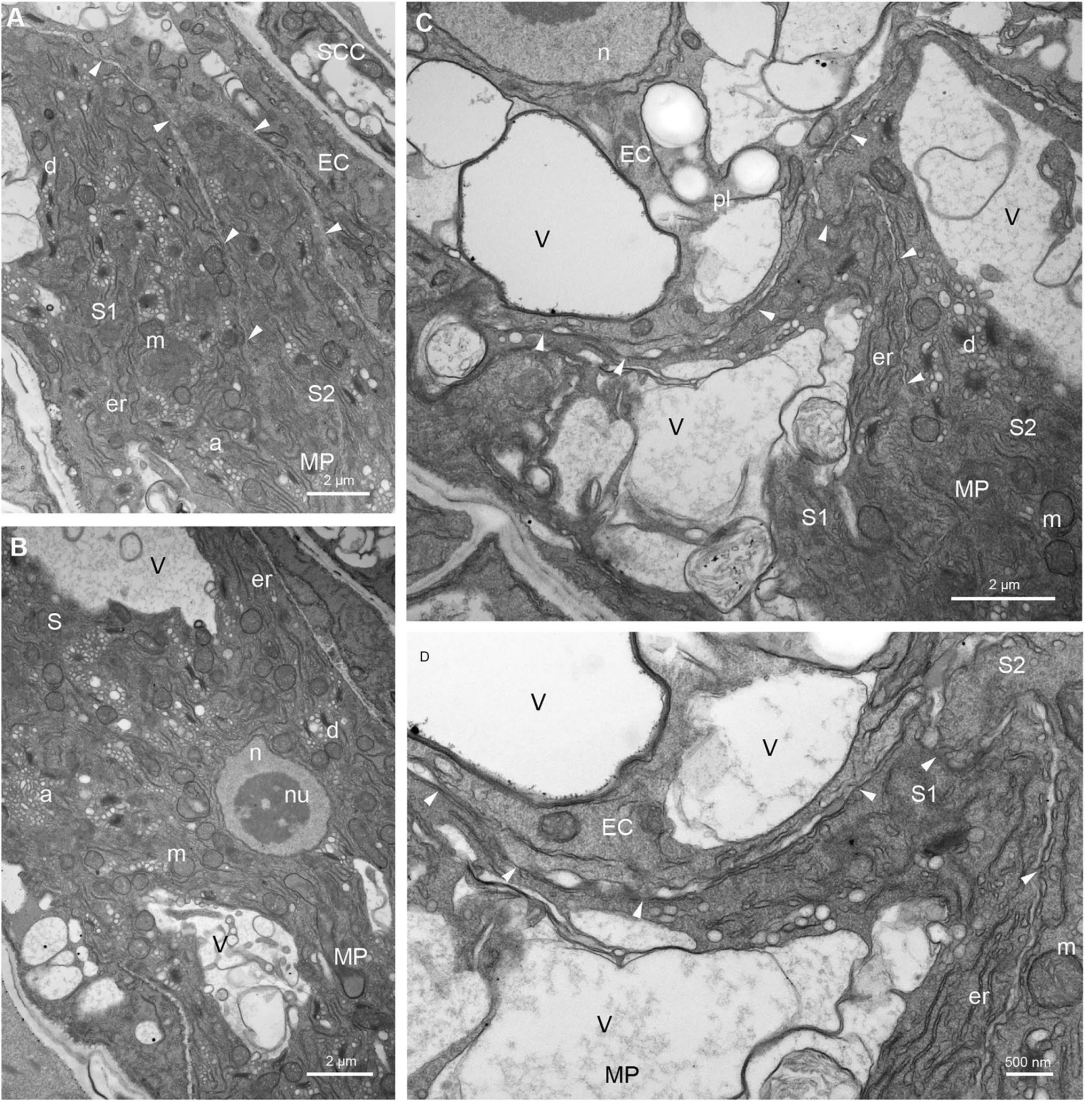
Transmission electron micrographs of serial sections of *A. thaliana* synergids. The cytoplasm of the Ss is rich in different organelles, such as mitochondria, dictyosomes, and extensive endoplasmic reticulum; numerous starch grains around the dictyosomes are also observed (**A**, **B**). The nucleus with a big nucleolus is localized in the center of cell (**B**). The cell wall separating both Ss and EC is irregular in shape, with numerous invaginations with areas of contact of plasma membranes (**A**, **C**, **D**, arrowheads). *MP* micropylar pole, *ChP* chalazal pole, *EC* egg cell, *S1, S2* synergids, *SCC* somatic cells, *er* endoplasmic reticulum, *d* dictyosomes, *m* mitochondrion, *a* amyloplasts, *V* vacuole, *pl* plastid, *n* nucleus, *nu* nucleolus

**Fig. 8:**
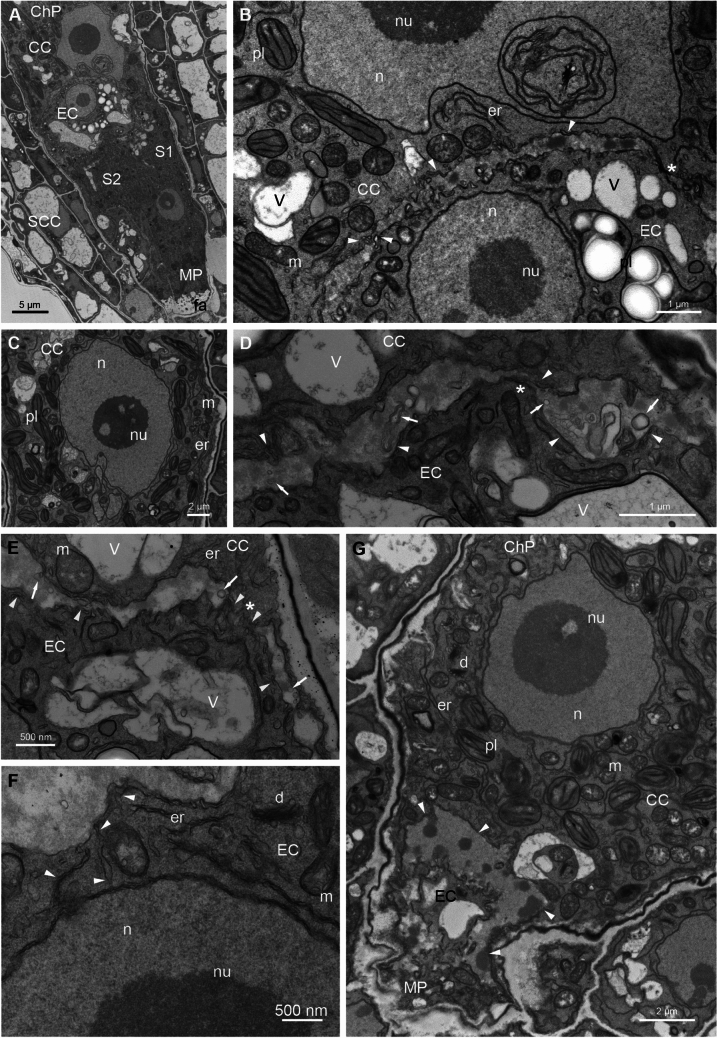
Transmission electron micrographs of *A. thaliana* embryo sac. In the longitudinal section, FGU is formed by elongated the Ss, EC, and CC (**A**). The nucleus of the CC with a big nucleolus is lobbed, and the numerous organelles, including many of the plastids, are visible in the cytoplasm (**B**, **C**, **G**). The EC nucleus is localized in the chalazal end of the cell, while the CC nucleus in the micropylar end of the cell is present (**B**). The EC and the CC border area is irregular and, in some places, discontinuous, allowing the plasma membranes to close contact (**B**, **D**, **E**, asterisks). Along the common of the EC and CC area, the numerous vesicles and cisternae of the endoplasmic reticulum are visible (**B**, **D**, **E**, arrowheads) sometimes they are elongated and located between the nucleus and the “cell wall?” (**F**, arrowheads). The EC and CC boundary cross sections show membrane structures, indicating the irregular structure of this area (**D**, **E**, arrow). The “cell wall” can be very thick in some places and contain patchy electron-dense material (G, arrowheads). *MP* micropylar pole, *ChP* chalazal pole, *EC* egg cell, *CC* central cell, *S1, S2* synergids, *SCC* somatic cells, *fa* filiform apparatus, *er* endoplasmic reticulum, *d* dictyosomes, *m* mitochondrion, *V* vacuole, *pl* plastid, *n* nucleus, *nu* nucleolus

Next, we analyzed the ultrastructure of *Arabidopsis* FGU using the en bloc staining method (Fig. 7, Fig. 8). In the micropylar region of the embryo sac, the two elongated Ss exhibit a cytoplasm rich in organelles (Fig. 7A, B). Among them, numerous mitochondria with well-defined cristae, dictyosomes, a few plastids containing starch grains, and an extensive endoplasmic reticulum can be observed. Additionally, numerous vesicles are present. A large vacuole is positioned at the chalazal end of the Ss, while small vacuoles are dispersed throughout the cytoplasm (Fig. 7C, D). The cell wall surrounding the Ss varies in thickness. At the micropylar end, a specialized cell wall structure known as a filiform apparatus is visible (Fig. 8A). Moving from the filiform apparatus toward the chalazal end of the Ss, the cell wall gradually thins and becomes irregular near the EC, with areas that stretch considerably or even disappear at some points. The cell wall separating the Ss is irregular in shape with numerous invaginations (Fig. 7A, Fig. 7C, D, arrowheads). The nucleus of the S with condensed chromatin and a nucleolus is centrally positioned between the vacuole and the filiform apparatus (Fig. 7B). In contrast to the Ss, the cytoplasm of the EC contains fewer organelles with numerous mitochondria, dictyosomes and endoplasmic reticulum which are distributed at the chalazal end of the cell. In the micropylar part of the EC, a large vacuole or small vacuoles of varying sizes are present (Fig. 8A). The nucleus with a big nucleolus is located at the chalazal end of the cell (Fig. 8A, B). En bloc staining reveals that the chromatin in this nucleus is decondensed. Plastids containing a few starch granules are positioned near the nucleus (Fig. 8B). The CC is the largest cell within the FGU and is where polar nuclei fusion occurs during maturation (Fig. 8C). The euchromatic nucleus with a large nucleolus and lobed structure is located in the micropylar region of the cell (Fig. 8A–C). The CC is highly vacuolated, containing numerous vacuoles, including a large central vacuole at the chalazal end (not shown). Most of the cytoplasm surrounding the nucleus is rich in numerous organelles, such as mitochondria, dictyosomes, plastids without starch granules, and extensive endoplasmic reticulum. The cytoplasm also contains many starch grains and lipid bodies (Fig. 8C, G). The EC shares a common boundary with the CC at the chalazal end, which is not defined as a typical cell wall as seen in somatic cells. Mansfield et al. (1990) described this boundary as “the gap between the EG and CC plasmalemmas.” In some areas, this boundary is irregular and discontinuous, bringing the plasma membranes in close contact (Fig. 8B, D–F, asterisk*s*). In some regions, “cell wall” extensions are present (Fig. 8G, arrowheads). In these regions, the accumulation of the electron-dense material is visible. Along this boundary, numerous vesicles and cisternae of endoplasmic reticulum are observed in both the EC and CC (Fig. 8B, D–F, arrowheads). Elongated cisternae of the endoplasmic reticulum extend from the irregular boundary to the EC nucleus (Fig. 8F, arrowheads). In the female gametes boundary sections, various membrane structures in different shapes are present, indicating the irregular structure of this area (Fig. 8D–E, arrows).

The nature of the boundary region between the EC and CC in the *A. thaliana* embryo sac remains poorly characterized. Methylene blue staining did not detect any staining in this area, in contrast to the staining observed in the somatic cell walls of the ovule (Fig. S4, arrowheads). However, occasional staining of cytoplasmic bridges was observed. Ultrastructural analyses indicate the presence of electron-dense material within this boundary region. To further investigate the composition of this extracellular matrix, we used the immunocytochemistry technique to localize specific components. We used the JIM7 antibody to detect methyl-esterified homogalacturonan, the LM19 antibody to identify unesterified homogalacturonan, and the LM5 antibody to localize β-(1,4)-galactan. Our results indicate a high pool of β-(1,4)-galactan and a lack/undetectable pool of the examined homogalacturonans. Additionally, Calcofluor White staining did not reveal the presence of cellulose in this area (Fig. S5).

## Discussion

During the maturation of *A. thaliana*, tricellular pollen grains undergo programmed dehydration, entering a metabolically quiescent state that enhances their resistance to environmental changes after anthesis (Moon and Jung 2020). Pollen is considered orthodox, meaning it is desiccation tolerant, remains viable and can germinate upon rehydration (Franchi et al. 2011; Firon et al. 2012). Ultrastructural analyses of dry pollen revealed the shrunken morphology of cells within the MGU, where dehydration is accompanied by poly(A)^+^RNA accumulation in the VN and the male gametes. In response to dehydration and to protect against hyperosmotic stress, mRNP particles form and accumulate within cytoplasmic/stress granules as part of a protective mechanism (Sorenson and Bailey-Serres 2014; Sze et al. 2021, 2024; Kubiak et al. 2023). The dehydration-induced condensation increases K^+^ ions concentration in the cytoplasm above the optimal level (100–150 mM), inhibiting mRNA binding to the ribosomes and the translation initiation (Bashe and Mascarenhas 1984). Pollen enters a phase of metabolic silencing. Upon rehydration, the pollen's endomembrane system rapidly recovers and the cellular metabolic processes are reactivated (Van Aelst et al. 1993; Yamamoto et al. 2003). We observed a homogenous distribution of poly(A)^+^RNA in both the cytoplasm and the nucleus of VC and SCs. The polyadenylated RNA stored in the granules is released, and the translation can be resumed (Hafidh et al. 2018; Sze et al. 2021). During germination, the K^+^ ion concentration reaches optimal levels for reinitiation of protein synthesis (Bashe and Mascarenhas 1984). The cytoplasmic projection of the SC connected to the VN is visible in our FISH analysis. The homogenous pattern of poly(A)^+^RNA localization is maintained within the MGU as it moves toward the tip of the in vitro growing pollen tube. Advances in single SC isolation techniques (Flores-Tornero and Becker 2023) have facilitated the identification of the SC transcriptome and genes (Borges et al. 2008, 2011; Slotkin et al. 2009; Misra et al. 2019, 2023). However, whether male gametes are transcriptionally active or silenced remains unresolved. On the other hand, it cannot be excluded that some of the transcripts identified in SCs could have been transported from the VC during pollen development (Jiang et al. 2015). Engel et al. (2005) demonstrated that in *A. thaliana* pollen, the promoter of *Gamete Expressed 1* (atGEX1) is active specifically in SCs but not in the GC or VC, indicating that the transcriptional activity of male gametes may be reactivated.

In contrast to the male gametophyte, the EC and the CC are enclosed within the embryo sac, surrounded by multiple layers of somatic cells of the ovule/ovary. The mature ovule is the site of fertilization and embryogenesis. The ultrastructure of cells within the FGU reflects their metabolic activity and biological function. The Ss are the most metabolically active within the FGU and are characterized by numerous organelles. We also observed the highest poly(A)^+^RNA levels in their cytoplasm and nuclei. In turn, mature female gametes accumulate a high pool of polyadenylated transcripts in their nuclei. The EC is transcriptionally silenced, whereas the CC remains active (Pillot et al. 2010). Whether, similar to the animal EC, this pool of poly(A)^+^RNA is a long-term RNA, and whether the progamic phase is a stage of “passive” waiting of the EC for SC remains an open question. Additionally, the timing of the zygotic genome activation (ZGA) in *A. thaliana* is still debated. Some data suggest that the zygote remains relatively quiescent, allowing the embryo to undergo several divisions without de novo transcription, while other data indicate that ZGA occurs soon after fertilization (Pillot et al. 2010; Nodine and Bartel 2012; Kao and Nodine 2019).

The distribution pattern of (poly)A^+^RNA in male and female gametes of *H. orientalis* differs from that in *A. thaliana*. In the SCs formed in the pollen tube of hyacinth, polyadenylated RNA is localized in the cytoplasmic granules, similar to the pattern observed in the dehydrated pollen grain of *A. thaliana.* Our previous studies revealed that *H. orientalis* SCs synthesize a new pool of RNA immediately after their formation although at a lower level than that observed in the VN. This transcriptional activity is accompanied by RNA polymerase II and a rich pool of splicing machinery elements. However, as the pollen tube continues to grow, RNA synthesis in the SCs nuclei is inhibited, and the splicing factors are gradually eliminated while the VN remains transcriptionally active (Zienkiewicz et al. 2011). This indicates that hyacinth SCs become metabolically silenced during pollen tube growth. In mature embryo sacs of *H. orientalis*, a high level of poly(A)^+^RNA is present only in the Ss, while in the transcriptionally silenced EC and CC (Niedojadło et al. 2012), the accumulation of polyadenylated transcripts in the cytoplasm and nucleus is very low. However, after fertilization, a drastic increase in poly(A)^+^RNA is observed in both the zygote and the primary endosperm cell (Pięciński et al. 2008). The different patterns of poly(A)^+^RNA distribution in these two species' SCs and female gametes may reflect distinct gamete development strategies. These differences may be attributed to the timing of the SCs formation, female gamete maturation, and the duration of the progamic phase. *Arabidopsis* has a short style, and fertilization occurs within 1–2 h after pollination (Christensen et al. 1997). In contrast, hyacinth has a longer pistil, with a style length of 2–3 mm, and the period between pollination and fertilization extends to 48 h or longer (Pięciński et al. 2008). To better understand these processes, future research on the MGU should include in vivo experiments.

The MGU is essential in the controlled transport and delivery of immotile SCs to the ovule/embryo sac (Russell 1984; Ge et al. 2011). The position of male gametes within the MGU is important in determining their migration in the growing pollen tube. In over 90% of *A. thaliana* pollen tubes, the VN precedes the SCs (Goto et al. 2020). Our observations indicate that within the MGU, SCs maintain close contact with the VN and are connected for movement through a cytoplasmic projection of one of the SCs. When the MGU enters the pollen tube, VN and SCs change to a slender shape, and the VN transport male gametes as passive cargo (Zhang et al. 2017). Disruption of MGU organization, such as exposure to elevated temperatures during in vitro pollen tube culture, leads to SCs separation and hinders their movement (Ge et al. 2011). The genetic control of the MGU assembly and positioning was revealed by Lalanne and Twell (2002). In *A. thaliana, GUM (germ unit malformed)* mutations disrupt the progressive anchoring of the VN and delay the MGU’s entry into the pollen tube, whereas *MUD* (*germ unit displaced*) mutations probably affect the cytoskeletal components anchoring the VN. The separation of the SCs from the VN and the displacement of the MGU to the pollen wall hinder the translocation of the unit into the pollen tube. Additionally, in *A. thaliana,* the migration sequence of VN and SCs, as well as subsequent pollen tube reception, is regulated by specific VN envelope proteins. The VN is highly invaginated, and KAKU4-dependent deformation facilitates its entry into the growing pollen tube and subsequent migration of the MGU (Goto et al. 2020). The movement of the MGU toward the tip of the elongating pollen tube is possible due to WPP domain-interacting proteins (WIPs) and their binding partners, WPP domain-interacting tail-anchored proteins (WITs), which generate a dual-driving force that moves the VN and SCs (Zhou and Meier 2014). A sperm cytoplasmic projection in the MGU remains intact during the pollen tube elongation. The physical connection of the sperm cells (SCs) and their enclosure within a shared inner membrane of the vegetative cell (VC), the peri-germ cell membrane (PGCM), are also crucial for the coordinated migration of the vegetative nucleus (VN) and male gametes as a unit (Sprunck 2020; Sugi et al. 2024).

The organization of the MGU and FGU provides intercellular communication between their elements (Mogensen 1992). Within the MGU, there are two possible pathways for transporting molecules from the VC to the male gametes. The first mechanism involves direct transport through the nuclear pores of the VN, which is closely associated with the long cytoplasmic projection of the SC. The second mechanism is indirectly from the VC cytoplasm via membrane-associated transport (Rauf et al. 2024). The *A. thaliana* VN is highly lobed; such a structure increases its surface area with numerous nuclear pores. The SCs are physically linked and enclosed by their plasma membrane and the peri-germ cell membrane, structures that become evident after hydration. A thin, non-fibrillar extracellular matrix devoid of plasmodesmata/plasmodesmata-like structures is present between these membranes. It is considered as an apoplast with unique features due to its origin (Sugi et al. 2024). Ultrastructural analysis of cross sections of the MGU in the pollen/pollen tube has also revealed irregular border between SCc and points of contact between the plasma membranes of both gametes. Although the precise molecular mechanisms of transport between the VC and SCs have not been elucidated so far, it is recognized that the MGU may act as a conduit for cell-to-cell communication. In *A. thaliana*, it has been proposed that TE-siRNAs (transposable element (TE)-derived small interfering RNAs) produced in the VN can migrate to the SCs to enhance TE silencing via the RdDM (RNA-directed DNA methylation) pathway in their genome or influence imprinted gene expression after fertilization (Slotkin et al. 2009; Grant-Downton et al. 2013; Creasey et al. 2014; Martinez et al. 2016). Additionally, the VC may contribute not only small RNA but also coding transcripts to the male gametes. Jiang et al. (2015) demonstrated that the AHG3 (ABA-hypersensitive germination3) gene, which encodes a protein phosphatase, is specifically transcribed in the *A. thaliana* VC but is predominantly translated in the SCs. Intercellular communication plays an important role in the functioning of the FGU. Small RNA can move freely within the FGU, from the CC to the EC (Ibarra et al. 2012; Erdmann et al. 2017), and can also induce non-cell-autonomous silencing from the Ss to the EC and the CC (Schröder et al. 2023). However, the precise molecular mechanism underlying this movement remains unknown. Our study investigated the unique structure of the boundary between the *A. thaliana* EC and CC. The extracellular matrix separating these both gametes is thick and irregular, with regions where cell membranes may come into contact. Erdmann et al. (2017) revealed that the CC and the egg apparatus remain symplastically connected after cellularization. Similar to the findings of Mansfield et al. (1991), we did not observe plasmodesmata, but we localized numerous membrane structures in cross sections. Whether these are potential channels/vesicles for the symplastic or apoplastic movement between cells remains an open question. Further research, including immunocytochemistry or FISH, is needed to localize specific molecules at the ultrastructure level.

Ultrastructural analysis of the extracellular matrix between the EC and CC in *A. thaliana* revealed a homogenous area devoid of fibrous elements and containing small masses of electron-dense material. These dense bodies are associated with microtubules to maintain the distance between cells and stabilize the boundary between cells during fertilization, ensuring that both female gametes remain in proximity to the SCs during recognition (Russell 1984; Huang and Russell 1992). The properties and the porosity of the cell wall are determined by its polysaccharide composition: cellulose, hemicellulose, and pectin matrix composed of homogalacturonan (HG), rhamnogalacturonan-I (RG-I), and rhamnogalacturonan-II (RG-II) that is often linked to HG (Ridley et al. 2001; Willats et al. 2001; Caffall and Mohnen 2009). Calcofluor White staining confirmed the lack of cellulose at the EC and CC boundary in *A. thaliana*. Immunolabeling using LM5 antibody revealed that the key element of the extracellular matrix of this area is β-(1,4)-galactan. This pectic polysaccharide is one of the main side chains of RG-I, which typically constitutes ∼20–35% of pectin with a backbone of alternating rhamnose and galacturonic acid residues, and side chains that include α−1,5-arabinans, β-(1,4)-galactans, and less frequently arabinogalactans (Silva et al. 2016). The role of pectic RG-I-associated LM5 β-(1,4)-galactan in cell walls remains unknown. Arabinans and galactans in RG-I are highly mobile and can interact, forming a transiently entangled matrix (Moore et al. 2008). The temporary presence of a pectic β-(1,4)-galactan epitope in cell walls has been observed to precede the main phase of cell elongation in the seedling root apex of *A. thaliana* (McCartney et al. 2003) and in etiolated hypocotyls and floral stem internodes (Moneo-Sánchez et al. 2019). This polymer may modulate cell wall properties essential for the transition to the rapid cell elongation phase. Similarly, in *A. thaliana*, the zygote elongates and polarizes before asymmetric cell division to generate a smaller apical and larger basal cell (Faure et al. 2002; Lau et al. 2012). Fertilization triggers modifications in the extracellular matrix of the EC and CC, with symplastic isolation occurring at later stages of embryo and endosperm development (Stadler et al. 2005; Ingram 2010; Erdmann et al. 2017). HG, the most abundant pectic polymer involved in forming calcium cross-linked gels that strengthen the network structure of the cell wall, was not detected in the *Arabidopsis* FGU. Methyl-esterified HG (JIM7) and unesterified HG (LM19) are probably present at low or undetectable levels. In contrast, in *H. orientalis*, both epitopes were localized in the EC and CC (Niedojadło et al. 2015a, b). The reason for these differences is not entirely clear, but it cannot be ruled out that they may be related to the time from fertilization to the first zygotic division. In *Arabidopsis*, this period is about 24 h (Kimata et al. 2016). The zygote initially measures only ∼20 µm in diameter but expands approximately ∼threefold along the apical-basal axis before dividing (Jürgens 2001). In the hyacinth, this process occurs over 48 h after fertilization, and the zygote elongates about ~ twofold. (Niedojadło et al. 2015b). This hypothesis requires further investigations including a detailed analysis of the structural and chemical composition of the extracellular matrix of female gametes of both species. Studying the distribution of a broader range of specific cell wall epitopes in the context of changes in the cell wall properties is necessary to better understand the potential for cell-to-cell communication and transport within the FGU before and after fertilization.

## Supplementary Information

Below is the link to the electronic supplementary material.

Fig. S1 Localization of poly(A)^+^RNA in *A. thaliana* (A-C) and H*yacinthus orientalis* L. (D-J) MGU in optical serial sections of in vitro growing pollen tube. In SCs of *A. thaliana*, the homogenous fluorescence signal in the cytoplasm and the interchromatin areas of the male gametes nuclei is observed, and the cytoplasmic projection (A-C, arrowheads) is visible. In VN, polyadenylated transcripts are also dispersed. In turn, in SCs of hyacinth (D-J) formed after the generative cell division in a growing pollen tube, polyadenylated transcripts are localized in granules in the cytoplasm (arrowheads), while in the VN, the signal of the fluorescence is homogenous. *VN* - vegetative nucleus, *SC1, SC2* - sperm cells, red - signal of FISH reaction, blue - Hoechst 33342 staining; A-C bars 5 µm, D-J bars 10 µm. Supplementary file1 (TIF 19802 KB)

Fig. S2 Digitally colored transmission electron micrographs of sections through MGU of dehydrated *A. thaliana* pollen grain. (A) corresponding to Fig. 3B and (B) corresponding to Fig. 3G. SCs are irregular in shape and form protrusions, which provide them with close contact with VN (B, arrowheads). *VC* - vegetative cell, *VN* - vegetative nucleus (blue – chromatin, red – nuclear envelope with numerous nuclear pores), SC1, SC2 - sperm cells (light green – chromatin, orange – nuclear envelope, yellow – sperm cell cytoplasm, pink - sperm cell plasma membrane, dark green – peri-germ cell membrane), *m* – mitochondrion. Supplementary file2 (TIF 24856 KB)

Fig. S3 Digitally colored transmission electron micrographs of sections through MGU of rehydrated *A. thaliana* pollen grain. (A) corresponding to Fig. 4E, (B) corresponding to Fig. 4F and (C) corresponding to Fig. 4L. After hydration, the close proximity of VN and SCs becomes more visible (B, arrowheads). The plasma membranes of SCs and VC are distinctly discernible (B-C, arrow), and interlocking cell borders connect SCs, which also share a common area filled with electron-dense material (C, asterisks). *VC* – vegetative cell, *VN* - vegetative nucleus (blue – chromatin, red – nuclear envelope with numerous nuclear pores), *SC1, SC2* – sperm cells (light green – chromatin, orange – nuclear envelope, yellow – sperm cell cytoplasm, pink - sperm cell plasma membrane, dark green – peri-germ cell membrane), *er* - endoplasmic reticulum, *d* - dictyosomes, *pl* - plastid, *m* - mitochondrion, *lb* - lipid body. Supplementary file3 (TIF 39924 KB)

Fig. S4 Structure of the *A. thaliana* FGU formed by the elongated Ss, EC, and CC. Longitudinal sections of the mature embryo sac after the methylene blue staining (A-C). The nucleus of the CC is located on the chalazal end of the cell, while the nucleus of the EC in the micropylar end is present. The border between the EC and CC without staining is visible (A-C, arrowheads). Sometimes, staining cytoplasmic bridges can be observed (B, arrows). *EC* - egg cell, *CC* - central cell, *S1, S2* – synergids, *SCC* - somatic cells; bars 10 µm. Supplementary file4 (TIF 19801 KB)

Fig. S5 Immunolocalization of methyl-esterified homogalacturonan (JIM 7 antibody) (A), unesterified homogalacturonan (LM19 antibody) (C), and β-(1,4)-galactan (LM5 antibody) (E) and Calcofluor White staining in the A. thaliana EC and CC. No labeling is observed for homogalacturonan detection using JIM7 and LM19 antibodies (A, C, arrowheads) and the cellulose staining (G, arrowheads), while a high pool of β-(1,4)-galactans is present. An intensive LM5 labeling is localized in the cell wall of the EC with Ss and in the border of the EC with CC (E, arrowheads). *EC* - egg cell, *CC* - central cell, *SCC* - somatic cells; A, C, E, - signal of the immunocytochemistry reaction, B, D, F - Dapi staining; bars 10 µm. Supplementary file5 (TIF 19802 KB)

ESM_1.mpg 3D confocal imaging of poly(A)^+^RNA localization in rehydrated *A. thaliana* pollen grain processed with Leica Application Suite Advanced Fluorescence 3.1.0 (LAS AF) software, red - signal of FISH reaction, blue – Hoechst 33342 staining. Supplementary file6 (MPG 2150 KB)

ESM_2.mpg 3D confocal imaging of poly(A)+RNA localization in *A. thaliana* pollen tube processed with Leica Application Suite Advanced Fluorescence 3.1.0 (LAS AF) software, red - signal of FISH reaction, blue – Hoechst 33342 staining. Supplementary file7 (MPG 736 KB)

## Data Availability

The data underlying this article will be shared upon reasonable request to the corresponding author.

## Abbreviations

GC: Generative cell
VC: Vegetative cell
SC: Sperm cell
EC: Egg cell
S: Synergid cell
CC: Central cell
A: Antipodal cell
