# Supplementary figures and images for: Bioimaging insights into structural pathways of cell-to-cell communication within the male (MGU) and female (FGU) germ units of Arabidopsis thaliana
Source: Plant Cell Rep. 2025 Feb 14;44(3):56. doi: 10.1007/s00299-025-03441-w (PMC11828830; doi:10.1007/s00299-025-03441-w)

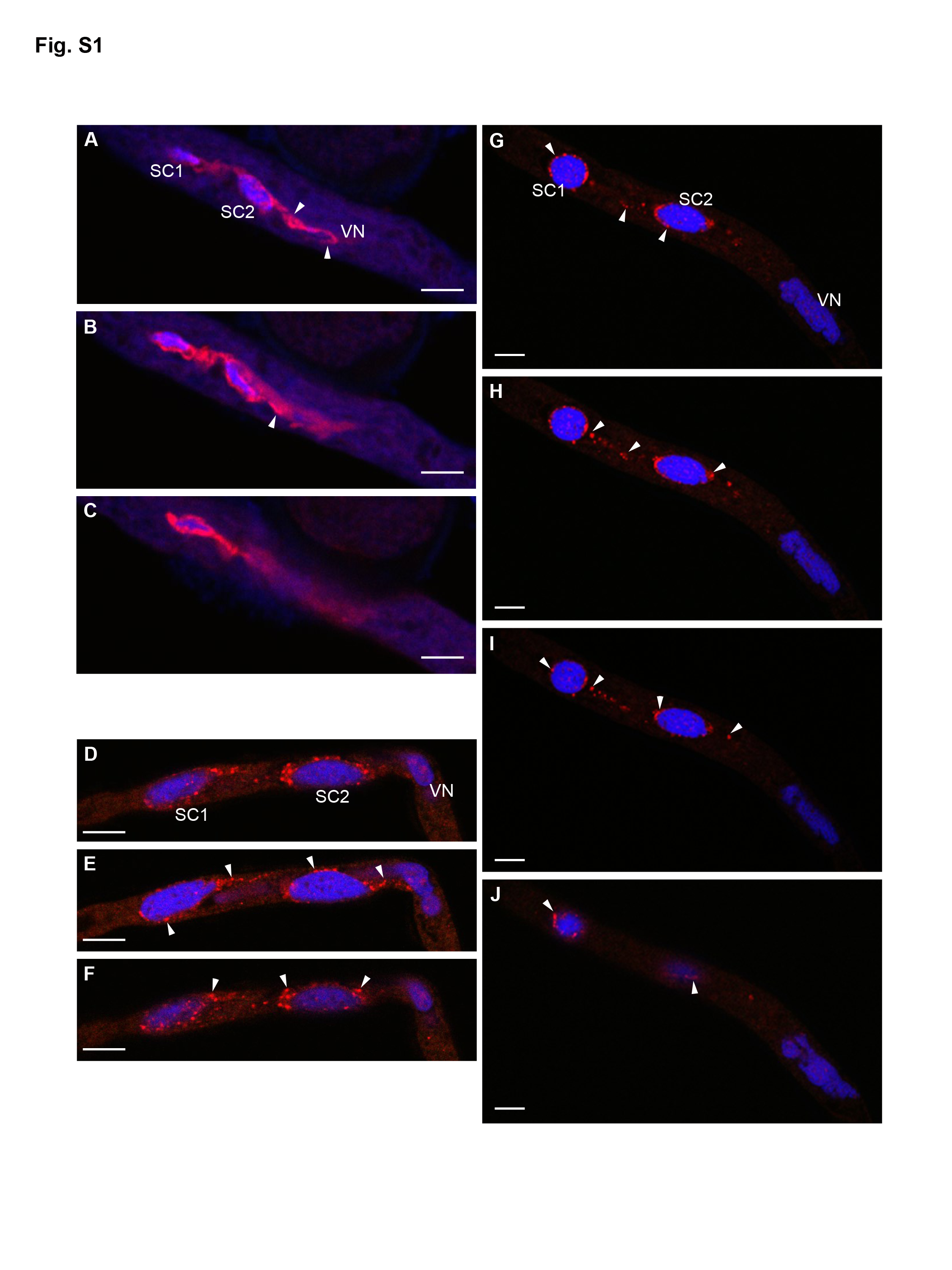

Supplement: Supplementary file 1 — Fig. S1 Localization of poly(A)+RNA in A. thaliana (A-C) and Hyacinthus orientalis L. (D-J) MGU in optical serial sections of in vitro growing pollen tube. In SCs of A. thaliana, the homogenous fluorescence signal in the cytoplasm and the interchromatin areas of the male gametes nuclei is observed, and the cytoplasmic projection (A-C, arrowheads) is visible. In VN, polyadenylated transcripts are also dispersed. In turn, in SCs of hyacinth (D-J) formed after the generative cell division in a growing pollen tube, polyadenylated transcripts are localized in granules in the cytoplasm (arrowheads), while in the VN, the signal of the fluorescence is homogenous. VN - vegetative nucleus, SC1, SC2 - sperm cells, red - signal of FISH reaction, blue - Hoechst 33342 staining; A-C bars 5 µm, D-J bars 10 µm. Supplementary file1 (TIF 19802 KB) [file 299_2025_3441_MOESM1_ESM.tif]

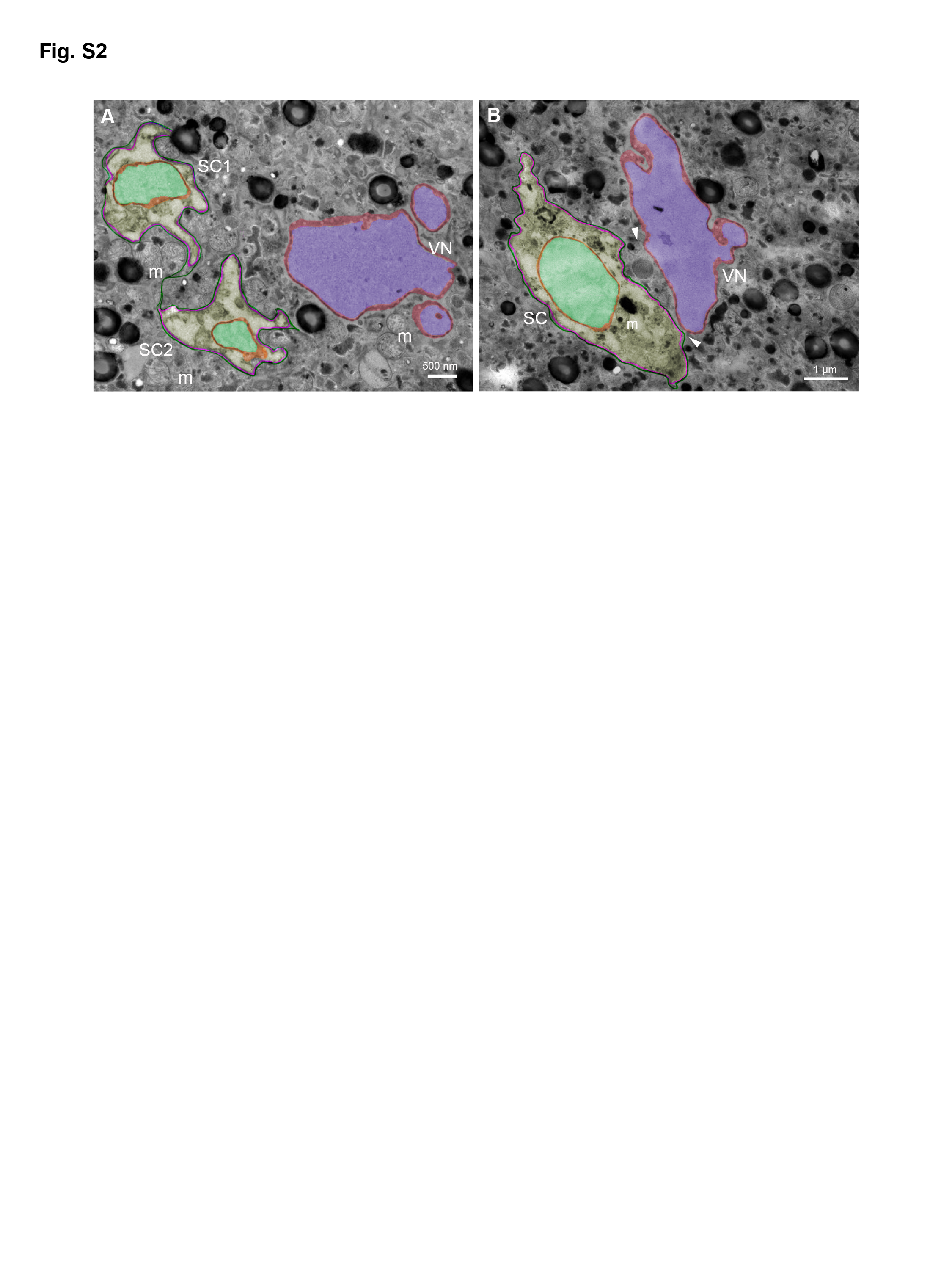

Supplement: Supplementary file 2 — Fig. S2 Digitally colored transmission electron micrographs of sections through MGU of dehydrated A. thaliana pollen grain. (A) corresponding to Fig. 3B and (B) corresponding to Fig. 3G. SCs are irregular in shape and form protrusions, which provide them with close contact with VN (B, arrowheads). VC - vegetative cell, VN - vegetative nucleus (blue – chromatin, red – nuclear envelope with numerous nuclear pores), SC1, SC2 - sperm cells (light green – chromatin, orange – nuclear envelope, yellow – sperm cell cytoplasm, pink - sperm cell plasma membrane, dark green – peri-germ cell membrane), m – mitochondrion. Supplementary file2 (TIF 24856 KB) [file 299_2025_3441_MOESM2_ESM.tif]

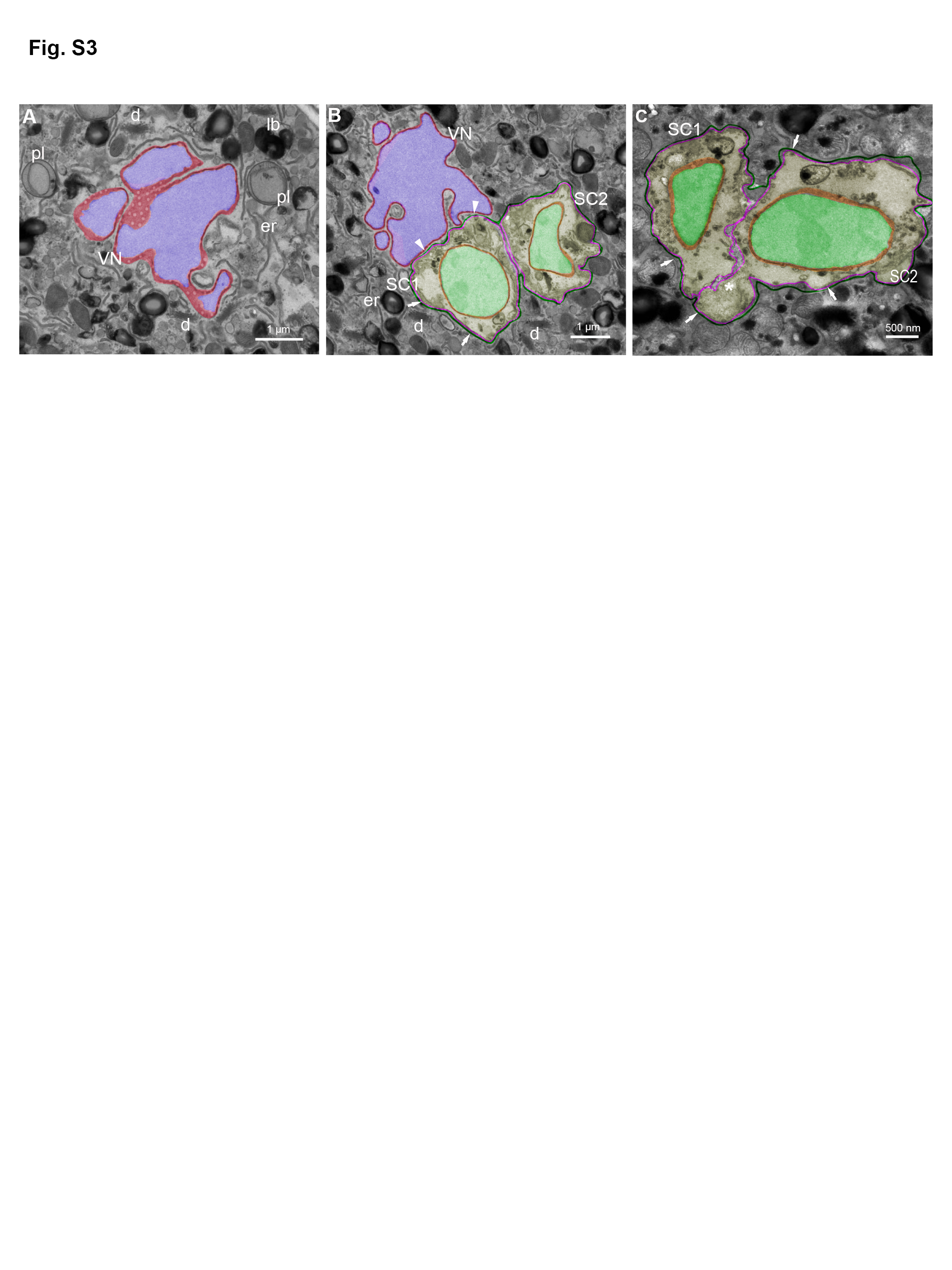

Supplement: Supplementary file 3 — Fig. S3 Digitally colored transmission electron micrographs of sections through MGU of rehydrated A. thaliana pollen grain. (A) corresponding to Fig. 4E, (B) corresponding to Fig. 4F and (C) corresponding to Fig. 4L. After hydration, the close proximity of VN and SCs becomes more visible (B, arrowheads). The plasma membranes of SCs and VC are distinctly discernible (B-C, arrow), and interlocking cell borders connect SCs, which also share a common area filled with electron-dense material (C, asterisks). VC – vegetative cell, VN - vegetative nucleus (blue – chromatin, red – nuclear envelope with numerous nuclear pores), SC1, SC2 – sperm cells (light green – chromatin, orange – nuclear envelope, yellow – sperm cell cytoplasm, pink - sperm cell plasma membrane, dark green – peri-germ cell membrane), er - endoplasmic reticulum, d - dictyosomes, pl - plastid, m - mitochondrion, lb - lipid body. Supplementary file3 (TIF 39924 KB) [file 299_2025_3441_MOESM3_ESM.tif]

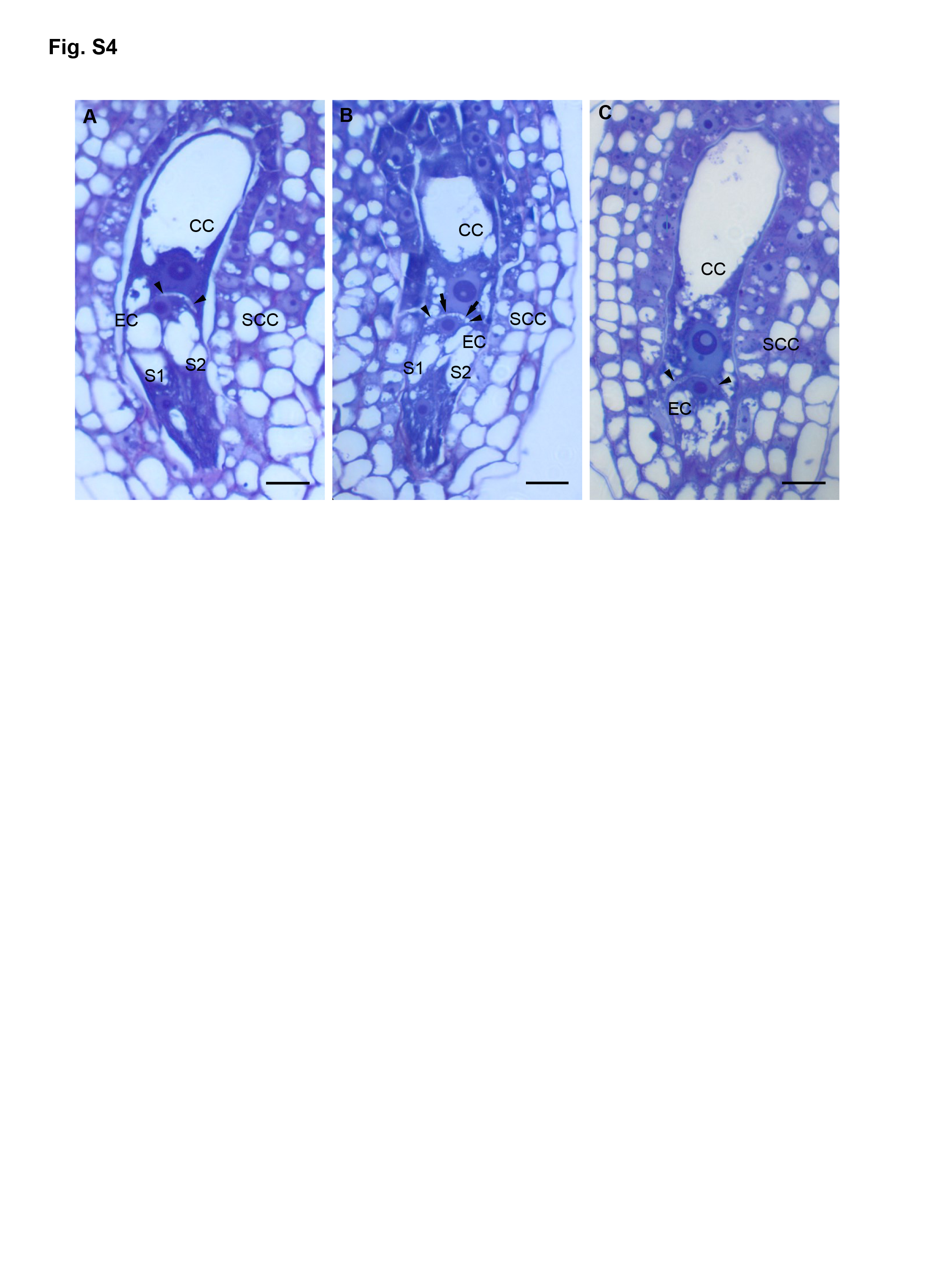

Supplement: Supplementary file 4 — Fig. S4 Structure of the A. thaliana FGU formed by the elongated Ss, EC, and CC. Longitudinal sections of the mature embryo sac after the methylene blue staining (A-C). The nucleus of the CC is located on the chalazal end of the cell, while the nucleus of the EC in the micropylar end is present. The border between the EC and CC without staining is visible (A-C, arrowheads). Sometimes, staining cytoplasmic bridges can be observed (B, arrows). EC - egg cell, CC - central cell, S1, S2 – synergids, SCC - somatic cells; bars 10 µm. Supplementary file4 (TIF 19801 KB) [file 299_2025_3441_MOESM4_ESM.tif]

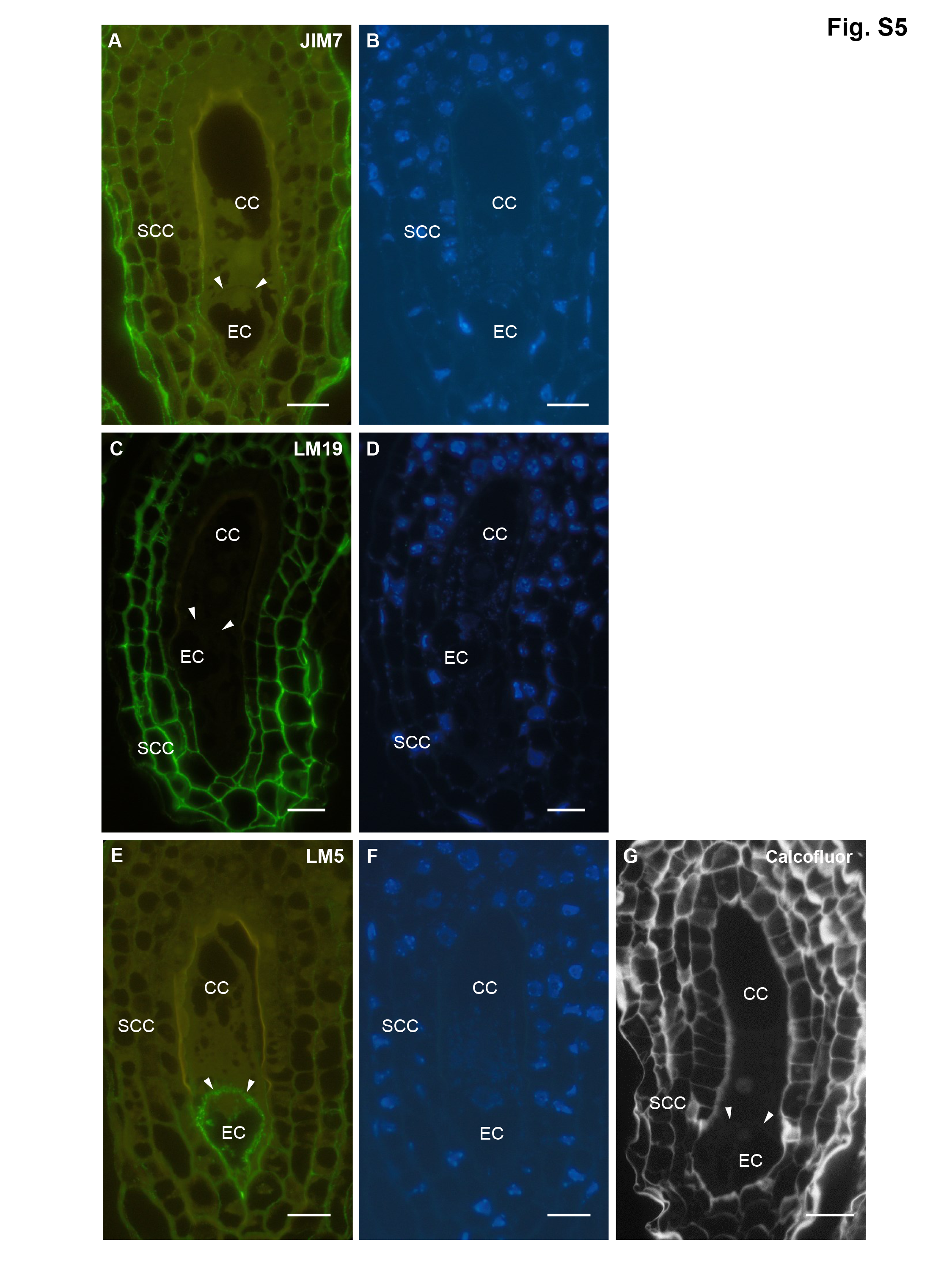

Supplement: Supplementary file 5 — Fig. S5 Immunolocalization of methyl-esterified homogalacturonan (JIM 7 antibody) (A), unesterified homogalacturonan (LM19 antibody) (C), and β-(1,4)-galactan (LM5 antibody) (E) and Calcofluor White staining in the A. thaliana EC and CC. No labeling is observed for homogalacturonan detection using JIM7 and LM19 antibodies (A, C, arrowheads) and the cellulose staining (G, arrowheads), while a high pool of β-(1,4)-galactans is present. An intensive LM5 labeling is localized in the cell wall of the EC with Ss and in the border of the EC with CC (E, arrowheads). EC - egg cell, CC - central cell, SCC - somatic cells; A, C, E, - signal of the immunocytochemistry reaction, B, D, F - Dapi staining; bars 10 µm. Supplementary file5 (TIF 19802 KB) [file 299_2025_3441_MOESM5_ESM.tif]
